# BAP1 Loss Affords Lipotoxicity Resistance in Uveal Melanoma

**DOI:** 10.1111/pcmr.70021

**Published:** 2025-04-29

**Authors:** C. J. Cunanan, A. Amirfallah, A. B. Sanders, K. C. Gallant, M. R. Cavallo, E. A. Homer, O. S. El Naggar, J. K. Farnan, G. Romano, J. L. Hope, J. G. Jackson, E. J. Hartsough

**Affiliations:** ^1^ Department of Pharmacology & Physiology Drexel University College of Medicine Philadelphia Pennsylvania USA; ^2^ Sidney Kimmel Comprehensive Cancer Center Philadelphia Pennsylvania USA; ^3^ Department of Microbiology & Immunology Drexel University College of Medicine Philadelphia Pennsylvania USA

**Keywords:** ferroptosis, lipid metabolism, lipids, lipotoxicity, liver, liver microenvironment, metastatic uveal melanoma, uveal melanoma

## Abstract

Uveal melanoma (UM) is an aggressive intraocular malignancy. Despite effective control of primary tumors, ~50% of UM patients develop metastases, with the liver being the predominant secondary site. BAP1 deficiency, present in ~80% of metastatic UM cases, is strongly associated with increased metastatic risk and poor prognosis. In silico analysis of UM patient samples suggests that reduced BAP1 is linked to enhanced expression of genes involved in fatty acid processing; therefore, we hypothesize that BAP1 deficiency primes UM cells for survival in the hepatic microenvironment by enhancing lipid tolerance and oxidative stress responses. Our findings demonstrate BAP1‐mutant UM resist lipotoxicity, whereas BAP1‐competent UM exhibit sensitivity due to lipid peroxide accumulation—a hallmark of ferroptotic‐like stress, and a response that can be mitigated by ferroptosis inhibition. Using an ex vivo liver slice model, we found that disrupting lipid metabolism with atorvastatin, an HMG‐CoA reductase inhibitor, reduced tumor burden of BAP1‐mutant UM. Moreover, we demonstrate a positive correlation between BAP1 and an epigenetic regulator of lipid homeostasis, ASXL2. Notably, ASXL2 depletion in BAP1‐competent UM phenocopies the lipotoxicity resistance observed in BAP1‐mutant UM—an effect that may be mediated by altered PPAR expression. This study reveals a novel mechanism linking BAP1 expression to lipid sensitivity via ASXL2, providing insights into liver tropism and potential therapeutic avenues for metastatic uveal melanoma.

## Graphical abstract

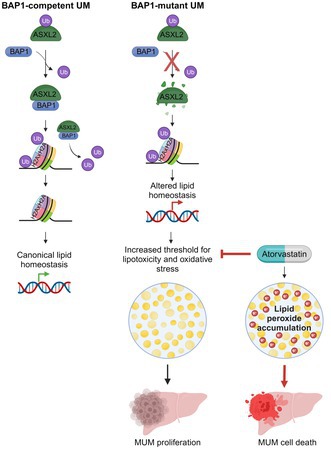

**Summary**

- BAP1 mutations occur in 80% of liver metastatic uveal melanomas (MUMs) and correlate with reduced survival, but how BAP1 loss promotes liver metastasis is not understood.
- This study elucidates a survival advantage in BAP1‐mutant UM and its translational implications.
- We show that BAP1‐mutant UM cells resist lipotoxicity and lipid peroxidation, an advantage that may be attributed to the dysregulation of the BAP1/ASXL2 axis.
- Using a novel ex vivo liver MUM model, we show that HMG‐CoA reductase inhibition with atorvastatin selectively reduces BAP1‐mutant UM tumor burden.
- These findings elucidate how BAP1‐mutant UM cells may adapt to survive in the lipid‐rich liver environment and identify a promising therapeutic target for liver MUM.

## 1 Introduction

Uveal melanoma (UM) is a rare and aggressive intraocular malignancy that arises in melanocytes of the uveal tract (Jager et al. 2020). Unlike many cancers, UM exhibits a low mutational burden, with mutations in the G(q) alpha subunit guanine nucleotide‐binding proteins, GNAQ or GNA11, presenting as the most common oncogenic drivers in primary lesions. These mutations limit their intrinsic GTPase activity, resulting in constitutive activation of downstream mitogenic pathways (Harbour 2012). While radiotherapy and enucleation are effective for primary UM tumors, approximately half of patients develop metastatic disease, predominantly to the liver (Carvajal et al. 2023; Rodriguez‐ Vidal et al. 2020).

Interestingly, 80% of liver metastatic UM (MUM) lesions harbor a deletion or inactivation in BRCA1‐associated protein 1 (BAP1), a nuclear deubiquitinase located on chromosome 3p21 (Harbour et al. 2010; Onken et al. 2006). BAP1 forms deubiquitinating complexes that modulate epigenetics, cell cycle regulation, DNA damage repair, metabolism, and apoptosis (Carbone et al. 2013). Interestingly, loss of BAP1 function has been implicated in the pathogenesis of various diseases, such as renal cell carcinoma, breast and lung cancers, and mesothelioma (Murali et al. 2013). However, the specific mechanisms by which BAP1 loss contributes to UM progression and metastasis remain largely unknown.

Among the many binding partners of BAP1 is additional sex combs‐like transcriptional regulator 2 (ASXL2). BAP1 and ASXL2 function as a complex to deubiquitinate histone H2A and mediate epigenetic regulation and chromatin remodeling (Daou et al. 2015). Notably, ASXL2 mediates lipid homeostasis, particularly through its association with peroxisome proliferator‐activated receptors (PPARs), transcription factors that modulate lipid metabolism and inflammation (Park et al. 2011; Izawa et al. 2015). However, the role of the BAP1/ASXL2 complex in BAP1‐mutant UM is not well understood.

The unique microenvironment of the liver may play a crucial role in the organotropism of BAP1‐mutant UM. As the primary organ for the storage and metabolism of fatty acids, the liver offers a lipid‐rich environment that may support the growth of metastatic UM cells (Li et al. 2020; Alves‐Bezerra and Cohen 2017; Ameer et al. 2014). The high bioavailability of lipids in the liver is significant in the context of liver metastases as polyunsaturated fatty acid metabolism is closely associated with ferroptosis, a form of regulated cell death characterized by the accumulation of lipid peroxides (Jiang et al. 2021). Given this interplay between the liver's lipid‐rich microenvironment and the role of fatty acid metabolism in ferroptosis, we hypothesize that BAP1 loss contributes to the successful metastasis of UM through unique management of the lipid‐rich liver microenvironment, thus providing insights to UM's hepatic organotropism.

Understanding the interactions between UM cells and the hepatic microenvironment is essential for developing novel treatment approaches. Currently, standard cancer therapies such as chemotherapy and immune checkpoint inhibitors have demonstrated limited efficacy in treating liver MUM, with patients given an overall survival of only 1 year, underscoring the need for improved therapeutic outcomes (Luke et al. 2015; Chua and Aplin 2018).

In this study, we illustrate a correlation between BAP1 deficiency and lipid tolerance. Our findings reveal that BAP1‐mutant UM cells are more resistant to lipotoxicity and lipid peroxidation compared to BAP1‐competent counterparts, providing a rationale for BAP1 loss and UM liver metastases. Importantly, we demonstrate that resistance to lipotoxicity associated with BAP1 loss is a targetable vulnerability as pharmacological inhibition of fatty acid metabolism and lipid ROS neutralization selectively impairs proliferation in BAP1‐mutant UM cells, highlighting a potential therapeutic strategy for liver MUM. Furthermore, we highlight how these responses may be linked to dysregulation of lipid homeostasis through loss of function of the BAP1/ASXL2 axis.

## 2 Methods

### 2.1 TCGA and GEO Analysis

Microarray data of uveal melanoma patient samples (7 BAP1‐wildtype and 12 BAP1‐mutant) were retrieved from the GEO database (accession: GSE78033). The BAP1 status of these samples was characterized in a previous study based on BAP1 mutations and chromosome 3 loss (Baqai et al. 2022). Differentially expressed genes were identified using the GEO2R tool. Gene expression data from 80 uveal melanoma patient samples (37 BAP1‐competent and 43 BAP1‐mutant) in the TCGA cohort were obtained and compared using the Xena browser and CBioPortal (Goldman et al. 2020). BAP1 status was determined using mutational status and copy number alterations; samples containing mutations and shallow or deep deletions were defined as “BAP1‐mutant” and those without mutations or copy number alterations were described as “BAP1‐competent”. Gene expression data for BAP1 mutant and wild‐type samples were obtained from UCSC Xena Browser. Differential gene expression analysis between these groups was performed using the Xena Differential Gene Expression Analysis tool (Table S1).

### 2.2 Enrichment Analysis

Enrichment analysis for biological functions and pathways associated with the upregulated DEGs was conducted using ClueGO (v.2.5.10), a plugin in Cytoscape (v.3.10.2) (Bindea et al. 2009; Shannon et al. 2003). The analysis utilized ontology and pathway databases, including GO: Biological Process (EBI‐UniProt‐GOA‐ACAP‐ARAP), KEGG, and Reactome Pathways, with all databases updated as of May 25, 2022. Only pathways with a significance level of *p* < 0.05 were considered. For enrichment analysis of commonly upregulated genes between GSE78033 and TCGA datasets, Enrichr (Chen et al. 2013) was utilized (Table S1).

### 2.3 Cell Culture

MP41, MP65, and MP46 cells were provided from Dr. Andrew Aplin (Thomas Jefferson University). 92.1(#13012458) and Mel202 (#13012457) cells were obtained from Sigma‐Aldrich. All cell lines are confirmed mycoplasma‐free. MP41, MP65, and MP46 cells were maintained in RPMI 1640 medium (Gibco) containing 20% FBS (Avantor Seradigm), 1%, 50 IU penicillin, and 50 μg/mL streptomycin (Gibco). 92.1 and Mel202 cells were maintained in RPMI 1640 medium (Gibco) containing 10% FBS (Avantor Seradigm), 1%, 50 IU penicillin, and 50 μg/mL streptomycin (Gibco).

### 2.4 Inhibitors, Exogenous Lipids, and Antibodies

Liproxstatin‐1 (#S7699), Z‐VAD‐FMK (#S7023), and Atorvastatin (#S5715) were purchased from Selleck Chemicals (Houston, TX). All inhibitors were dissolved in DMSO. LA (#L1012) and cis‐4,7,10,13,16,19‐Docosahexaenoic acid (#D2534) were purchased from Millipore Sigma. Exogenous lipids were dissolved in a 20% BSA/100% ethanol solution. AIFM2/FSP1 (#2497 L10122), GPX4 (#52455), LCN2 (#44058), GAPDH (#2118), Tubulin (#2148), BAP1 (#13271), ASXL2 (#71257), Ubiquityl‐Histone H2A (#8240), and Histone H2A (#12349) were purchased from Cell Signaling Technology. PPARα (#398394) was purchased from Santa Cruz Biotechnology. PPARγ (#PA52575) was purchased from Thermo Fisher Scientific. PE‐conjugated anti‐mouse CD31 (#102408) was purchased from BioLegend.

### 2.5 Cell Proliferation Assay

Cells were seeded at 5.0 × 10^4^ per well in 6‐well plates in culture medium overnight. The next day, the medium was supplemented with drugs of interest. For rescue experiments, cells were pre‐treated with Liproxstatin‐1 or Z‐VAD‐FMK for 24 h before lipid treatment. Medium and drugs were renewed every 2 days for 6 days. At endpoint, cells were rinsed with 1X PBS, then fixed and stained in buffered formalin with 0.2% crystal violet. Plate coverage was quantified using ImageJ.

### 2.6 Western Blotting

For western blot analysis of baseline expression, 2.0 × 10^5^ cells were seeded in 6‐well plates in culture medium overnight and lysates were collected the next day. For western blot analysis of inducible cell lines, 1.0 × 10^5^ cells were seeded in 6‐well plates in culture medium overnight. Cells were then treated with 2 mg/mL doxycycline and lysates were collected after 24–48 h of doxycycline treatment. Cell lysates were harvested in 1X Laemmli buffer and 5% BME. SDS‐PAGE gels were loaded with 15 μg of protein per lane and transferred to PVDF membranes via TransBlot Turbo System (BioRad Laboratories). Membranes were blocked in 1% BSA in PBS containing 0.1% Tween (PBST) for 1 h at room temperature. All primary antibodies were brought up in 1% BSA/PBST and incubated overnight at 4°C. The following day, membranes were washed three times for 15 min each in PBST before incubating in secondary HRP antibodies brought up in 5% nonfat dry milk/PBST. Western blots were developed in SuperSignal West Pico Chemiluminescent Substrate (Thermo Scientific) using the ChemiDoc XRS+ imaging system and Quantity One imaging software (BioRad Laboratories). Densitometry analysis was conducted in ImageJ.

### 2.7 Lipid Peroxidation Measurements

To measure lipid peroxidation under exogenous lipid treatment, 7.5 × 10^4^ cells were seeded in 6 well plates and cultured overnight. Cells were then treated with 200 μM LA (for 24 h), 150 μM DHA (for 96 h), or 25 and 50 μM Atorvastatin (for 96 h). For rescue experiments, cells were pre‐treated with Liproxstatin‐1 for 24 h before lipid treatment. Cells were stained with 3 μM Bodipy 581/591 C11 Lipid Peroxidation Sensor (Thermo Fisher Scientific #D386) in cell culture medium for 30 min at 37°C. Stained cells were then fixed with 1% PFA, analyzed on CytoFLEX Flow Cytometer (Beckman Coulter Inc.), and data were analyzed with FlowJo.

### 2.8 Lentiviral Cloning and Stable Cell Lines

Human BAP1 was amplified from cDNA (Dharmacon Clone ID: 3543914) cloned into pENTR/D‐TOPO entry vectors (Invitrogen), and recombined into pLIX_403 (Addgene #41395) using LR Clonase II (Invitrogen). 3 μg of pLIX_403/BAP1 and appropriate lentiviral packaging plasmids were transfected into LentiX‐293t cells (Takara) for lentiviral production using FuGENE 6 Transfection reagent (Promega). Viral particles were precipitated after 72 h and used to transduce MP46 cells for inducible expression under doxycycline control. MP46 cells were treated with puromycin for antibiotic selection 72 h post‐transduction for 2 weeks. Puromycin‐resistant clones were isolated and used for analysis.

### 2.9 siRNA Transfection

Cells were seeded at 7.5 × 10^4^ per well and transiently transfected with Dharmacon siRNAs at a final concentration of 25 nmol/L using Lipofectamine RNAiMAX (Invitrogen). Nontargeting control (5′‐UGGUUUACAUGUCGACUAA‐3′) or ASXL2 (J‐022638‐11‐0005, J‐022638‐12‐0005) siRNAs were incubated with RNAiMAX in OptiMEM (Gibco) at RT for 15 min before administering to cells. Transfection was incubated for 4 h at 37°C with 5% CO_2_. Serum containing media was added to the transfection cocktail and incubated for 72 h before experimentation.

### 2.10 Intrasplenic Injection Surgery

8–10 week old male NOD.Cg‐Prkdc^scid^ Il2rg^tm1Wjl^/SzJ (NSG) mice (The Jackson Laboratory, Strain #005557) were injected with MP46 cells as previously described (Sugase et al. 2020). Briefly, mice were positioned in the right lateral recumbent position. A 1 cm incision was made in the left upper abdominal wall, and a 1 cm incision in the peritoneum was made to expose the spleen. 2.0 × 10^6^ GFP luciferase‐expressing MP46 cells in 50 μL supplemented RPMI 1640 medium (Gibco) were injected into the spleen. The injection site of the needle was cauterized to limit bleeding. 15 min after injection, a splenectomy was performed using a surgical cautery tip. The peritoneum incision was closed with absorbable 5‐0 polydioxanone sutures, and the abdominal wall incision was closed with 3‐0 silk sutures. 3 days after injection, body weight and tumor burden were monitored biweekly via bioluminescent imaging for 3 weeks after injection. After 3 weeks, organotypic liver slice cultures were harvested. This animal study was approved by the Institutional Animal Care and Use Committee of Drexel University and adhered to the recommendations in the National Institutes of Health Guide for the Care and Use of Laboratory Animals.

### 2.11 Bioluminescent Imaging

For in vivo studies, mice were injected intraperitoneally with 150 mg/kg of IVISBright D‐Luciferin (Revvity #122799) and images were quantified using Living Image software (Caliper Life Sciences, Waltham, MA, United States). For ex vivo studies, samples were treated with 150 μg/mL IVISBright D‐Luciferin, and images were quantified using Living Image software (Caliper Life Sciences, Waltham, MA, United States).

### 2.12 Organotypic Liver Slice Cultures

8–10 week old male tumor‐bearing NSG mice were anesthetized with 3% isoflurane for induction and 2% for maintenance. Mice were placed on a dissection tray in the supine position, and limbs were secured using surgical tape. The abdomen was sterilized with 70% ethanol. A celiotomy was performed by making a 3 cm incision on the abdominal wall and the peritoneum. The vena cava was cannulated with a 25‐gauge catheter. Manual perfusion of 1X PBS began after cannulation of the vena cava at a flow rate of 1 mL per minute. After 20 s of perfusion, the portal vein was cut with dissection scissors to release blood from the portal vein and liver. Efficient perfusion was confirmed by swelling of the liver before cutting the portal vein and pale color change of each lobe after cutting the portal vein. The liver was perfused with a total volume of 5 mL of 1X PBS. At the conclusion of the perfusion, the liver was dissected, and 250 μm slices were harvested using a Compresstome vibrating microtome (Precisionary Instruments). 2–3 liver slices were placed onto each 0.4 μm Millicell tissue culture insert (Millipore) in six‐well plates, with 1 mL of RPMI 1640 medium (Gibco) containing 10% FBS (Avantor Seradigm), 1%, 50 IU penicillin, and 50 μg/mL streptomycin (Gibco). Liver slices containing UM via intrasplenic injection were treated with the drug of interest for 0–72 h, and tumor growth was measured via bioluminescent imaging.

### 2.13 Immunofluorescence Microscopy of Organotypic Liver Slice Cultures

Organotypic liver slice cultures were fixed with 4% PFA and optically cleared as previously described (Hsu et al. 2022). Briefly, slices were incubated with 50% (*v*/*v*) tetrahydrofuran (with 250 ppm BHT, Millipore‐Sigma, #186562) in sterile Milli‐Q water on an orbital shaker in a vented chemical fume hood for 48 h at room temperature. Slices were then washed four times in Milli‐Q water for 1 h. Following optical clearing, slices were permeabilized with 0.4% Triton X‐100 and stained overnight with PE‐conjugated anti‐mouse CD31. The following day, slice cultures were washed 3 times with 1X PBS and mounted on glass slides with a mounting solution of 80% Nycodenz (Accurate Chemical & Scientific #100334‐594), 7 M urea, and 0.05% sodium azide prepared in 0.02 M sodium phosphate buffer. Slides were incubated in mounting solution for 24 h, and images were obtained using a 10× and 30× objective on an Olympus Fluoview FV3000 confocal microscope.

### 2.14 ATP Viability Assay

ATP levels of 2–3 mg of liver tissue were measured using the CellTiter‐Glo 3D Cell Viability Assay (Promega, #G9681) according to the product protocol.

### 2.15 Statistical Analysis

Unless noted otherwise, experiments were repeated at least 3 times and significance was determined by one‐way or two‐way ANOVA analysis with post hoc Tukey HSD test using GraphPad Prism 10. Data represent mean values and error bars denote the standard error of the mean (SEM). Unless specified, ns is indicative of *p* > 0.05, * of *p* < 0.05, ** of *p* < 0.01, *** of *p* < 0.001, and # *p* < 0.0001.

## 3 Results

### 3.1 Transcriptional Profiling Reveals Upregulation of Fatty Acid Metabolism in BAP1‐Mutant Uveal Melanoma

**FIGURE 1:**
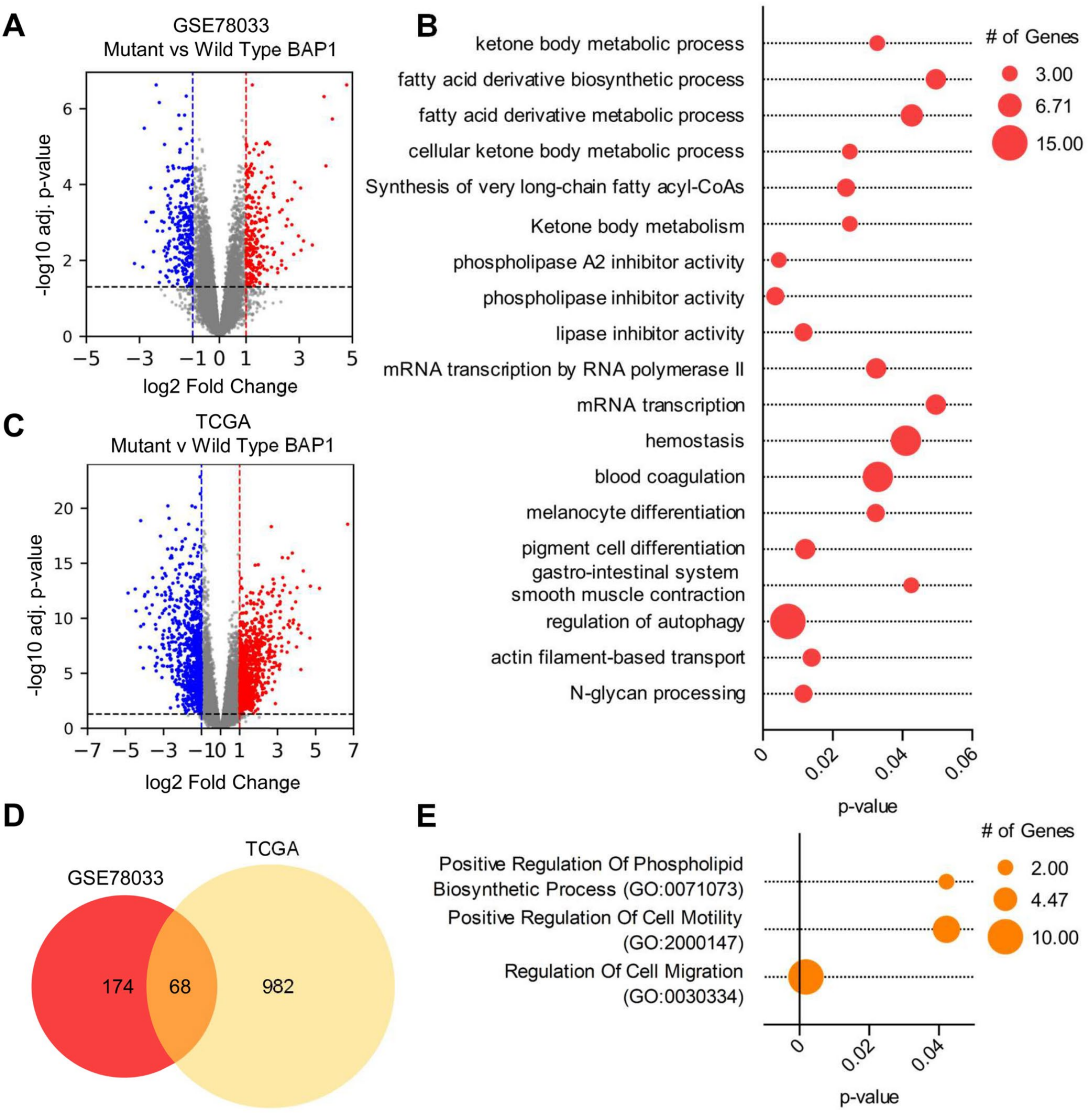
BAP1‐mutant UM potation exhibits an enhanced fatty acid metabolism signature. (A) Volcano plot analysis of differentially expressed genes comparing BAP1‐mutant versus BAP1‐competent UM patient samples from GSE78033 dataset. Red dots indicate significantly upregulated genes, and blue dots indicate significantly downregulated genes (adjusted *p‐value* < 0.05, |log2 fold change| > 1). (B) Pathway enrichment analysis of significantly upregulated genes in BAP1‐mutant samples using ClueGO. Circle size corresponds to gene count within each pathway (3.00–15.00 genes), and statistical significance is shown on the *x*‐axis. (C) Volcano plot analysis of differential gene expression between BAP1‐mutant and BAP1‐competent samples from TCGA uveal melanoma dataset. (D) Venn diagram showing the overlap of significantly upregulated genes between GSE78033 and TCGA datasets, identifying 68 commonly dysregulated genes. (E) Pathway analysis of the 68 shared genes; pathways shown have adjusted *p‐value* < 0.05. Circle size indicates gene count (2.00–10.00 genes), and statistical significance is shown on the *x*‐axis.

To investigate molecular differences between BAP1‐mutant and BAP1‐competent UM, we analyzed differential gene expression using the GSE78033 dataset derived from patient xenografts (Laurent et al. 2013). Our analysis identified distinct transcriptional profiles between these groups, visualized in a volcano plot showing both upregulated and downregulated genes in BAP1‐mutant samples (Figure 1A). To understand the functional implications of these expression changes, we performed pathway enrichment analysis on significantly upregulated genes (adjusted *p‐value* < 0.05, log2 fold change > 1) using ClueGO (Table S1). This analysis revealed several enriched pathways related to lipid metabolism (Figure 1B). Notably, of the 19 enriched pathways, 9 relate to lipid metabolism and phospholipase activity, suggesting altered lipid metabolism in BAP1‐mutant UM samples. We next questioned if the upregulated genes within the lipid metabolism and phospholipase activity‐associated pathways are also upregulated in mutant samples within the TCGA dataset (Table S1). Here, we separated BAP1‐competent and BAP1‐mutant patient samples and assayed transcript levels of the 11 genes found in the upregulated lipid metabolism and phospholipase activity pathways identified in Figure 1B: ASCL1, ACSS3, HMGCL, OXCT1, HACD1, HACD4, HSD17B12, ANXA1, ANXA2, ANXAP2, and ANXA4 (Figure S1). Of these 11 targets identified in the GSE78033 dataset, 10 genes are significantly increased in the BAP1‐mutant UM TCGA cohort, providing further evidence linking BAP1 loss of function to altered lipid metabolism.

To further validate these findings, we analyzed UM samples from TCGA, comparing BAP1‐mutant versus competent UM profiles (Figure 1C) (adjusted *p‐value* < 0.05, log2 fold change > 1). The intersection of differentially expressed genes between GSE78033 and TCGA datasets revealed 68 commonly upregulated genes (Figure 1D), and gene ontology analysis of these shared targets highlighted significant enrichment in pathways related to phospholipid biosynthesis, cell motility, and migration (Figure 1E).

These analyses reveal a previously unrecognized association between BAP1 mutation status and altered lipid metabolism in UM. The consistent enrichment of lipid‐related pathways suggests that modified lipid metabolism may be a fundamental characteristic of BAP1‐mutant UM. While our analysis also identified enrichment in cell motility and migration pathways (Figure 1E), these aspects have been recently characterized (Baqai et al. 2022). Therefore, we focused our investigation on the novel finding of dysregulated lipid metabolism, which may contribute to the distinct clinical behavior and metastatic propensity of BAP1‐mutant uveal melanoma.

### 3.2 BAP1‐Mutant UM Cells Are Insensitive to Lipotoxicity and Lipid Peroxidation

**FIGURE 2:**
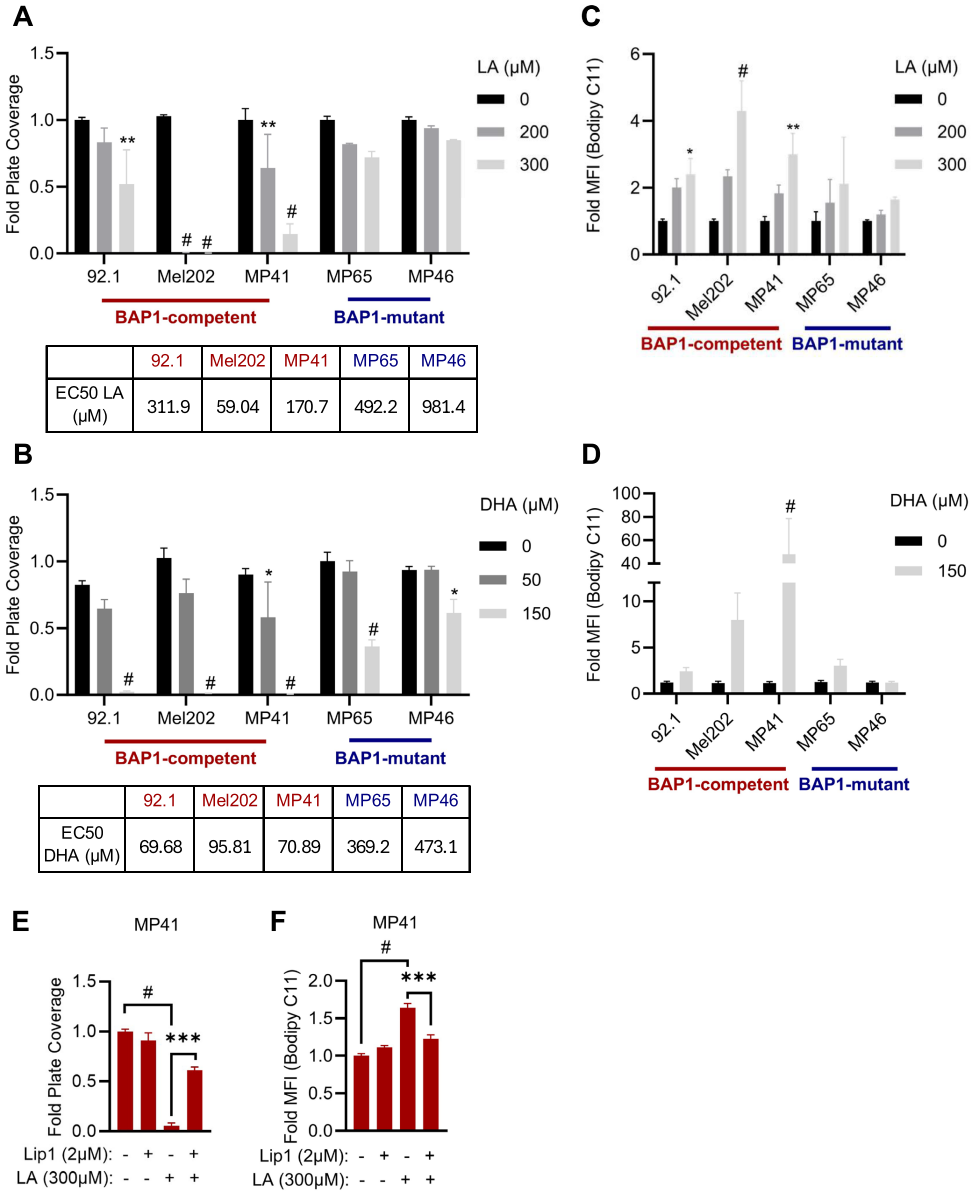
BAP1‐mutant UM are more resistant to lipotoxicity and lipid peroxidation. (A, B) Cell proliferation indicated by crystal violet staining and corresponding EC50s of 92.1, Mel202, MP41, MP65, and MP46 cells treated with increasing levels of linoleic acid (LA) (0, 200, and 300 μM—A) or docosahexaenoic acid (DHA) (0, 50, and 150 μM—B). Full dose curves, representative images, and summary tables of significance between BAP1 competent versus mutant cell lines can be found in Figures S2 and S3. (C, D). Lipid peroxidation levels indicated by Bodipy 493/593 C11 staining of 92.1, Mel202, MP41, MP65, and MP46 cells treated with 0, 200, or 300 μM LA for 24 h (C) and 0, 150, or 300 μM DHA for 96 h (D). Summary tables of significance between BAP1 competent versus mutant cell lines can be found in Figure S4. (E, F) Cell proliferation indicated by crystal violet staining (E) and lipid peroxidation levels indicated by Bodipy 493/593 C11 (F) of MP41 cells treated with 2 μM Lip1, 300 μM LA, or both. The quantification of crystal violet staining is represented by fold plate coverage, and Bodipy 493/593 C11 staining by fold MFI compared to vehicle treatment. Results are the averages from at least three independent repeated experiments. The * is indicative of *p* < 0.05, ** of *p* < 0.01, *** of *p* < 0.001, and # *p* < 0.0001 as determined by two‐way ANOVA analysis with multiple comparisons (A–D) or one‐way ANOVA (E, F).

Since the analysis of BAP1‐mutant UM patient samples revealed the enrichment of pathways associated with fatty acid metabolism, we hypothesized that BAP1‐competent and BAP1‐mutant UM cells might respond differently to the lipid‐rich hepatic microenvironment. To test this, we measured the proliferative capacity of five UM cell lines—three BAP1‐competent (92.1, Mel202, and MP41) and two BAP1‐mutant (MP65 and MP46), treated with increasing concentrations of polyunsaturated fatty acids (PUFA) primarily found in the liver. UM cells dosed with exogenous PUFA were incubated for 6 days; at endpoint, crystal violet staining demonstrated that BAP1‐mutant cells exhibited significantly higher tolerance to elevated levels of linoleic acid (LA) compared to BAP1‐competent cells (Figures 2A, S2). We observed similar results when cells were treated with docosahexaenoic acid (DHA), another PUFA abundant in the liver (Figures 2B, S3) (Jump et al. 2008; Paul et al. 2022).

Given that high rates of PUFA oxidation can result in toxic levels of lipid peroxides, a hallmark of ferroptotic stress (Jiang et al. 2021), we next quantified lipid peroxidation levels in BAP1‐competent and ‐mutant UM cell lines using BODIPY 493/503 C11 staining. Cells were exposed to LA or DHA at concentrations previously shown to inhibit proliferation (Figure 2A,B), and we found that BAP1‐competent UM had significantly higher lipid peroxide levels compared to their BAP1‐mutant counterparts (Figures 2C,D, S4A,B). We next sought to confirm if these observations are linked to ferroptotic activity. Treatment with a radical‐trapping antioxidant and ferroptosis inhibitor, liproxstatin‐1 (Lip1) (Zilka et al. 2017), significantly rescued the LA‐associated sensitivity in the BAP1‐competent MP41 cells (Figure 2E). Consistent with this, Lip1 treatment reduced LA‐mediated lipid peroxidation (Figure 2F). These effects were largely mirrored in a second BAP1‐competent cell line, 92.1, where Lip1 rescued proliferation and suppressed lipid peroxidation associated with LA treatment (*p* values of 0.089 and 0.076, respectively) (Figure S4C,D). Importantly, the broad‐spectrum caspase inhibitor Z‐VAD‐FMK failed to rescue these effects, providing further evidence of ferroptotic‐like stress (Figure S5). Taken together, this data demonstrates that, unlike mutant BAP1 UM, BAP1‐competent UM cells are sensitive to ferroptotic‐like stress/lipotoxicity.

### 3.3 BAP1‐Mutant UM Exhibit Sensitivity to Lipid Metabolism Inhibition In Vitro and Ex Vivo

**FIGURE 3:**
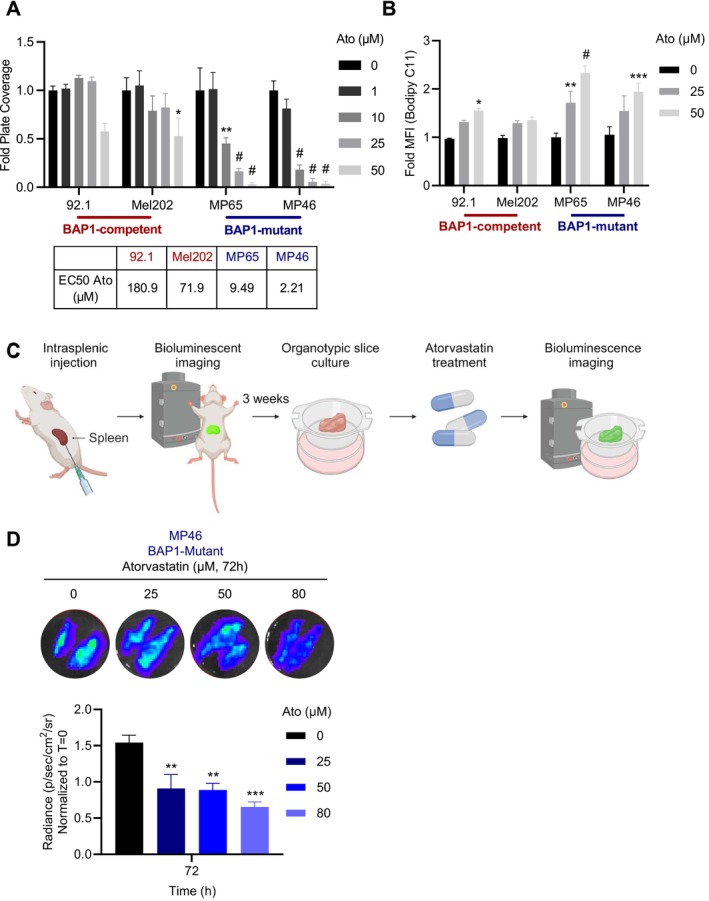
BAP1‐mutant UM are sensitive to inhibition of lipid metabolism in vitro and in an ex vivo liver MUM model. (A) Cell proliferation indicated by crystal violet staining and corresponding EC50s of 92.1, Mel202, MP65, and MP46 cells treated with increasing levels of atorvastatin (0, 1, 10, 25, and 50 μM) for 6 days. Full dose curves, representative images, and summary tables of significance between BAP1 competent versus mutant cell lines can be found in Figure S6A–C. (B) Lipid peroxidation levels indicated by Bodipy 493/593 C11 staining of 92.1, Mel202, MP65, and MP46 cells treated with increasing levels of atorvastatin (0, 25, and 50 μM) for 96 h. Summary tables of significance between BAP1 competent versus mutant cell lines can be found in Figure S6D. (C) Schematic representation of the workflow for generating an ex vivo model of BAP1‐mutant liver metastatic uveal melanoma (UM). (D) Representative images depicting BAP1‐mutant (MP46) tumor growth detected by bioluminescence in organotypic liver slices treated with increasing concentrations of atorvastatin (0, 25, 50, and 80 μM) after 72 h. The quantification of tumor burden is represented by fold bioluminescence compared to vehicle treatment at 0 h. Quantification of crystal violet staining is represented by fold plate coverage, and Bodipy 493/593 C11 staining by fold MFI compared to vehicle treatment. Results are the averages from at least three independent repeated experiments, and ex vivo slice experiments are the averages of 3 slices per mouse and *n* = 3–4 mice per group. The * is indicative of *p* < 0.05, ** of *p* < 0.01, *** of *p* < 0.001, and # *p* < 0.0001 as determined by one‐way (D) and two‐way ANOVA (A, B) analysis with multiple comparisons.

Given that BAP1‐mutant cells are more resistant to lipotoxicity compared to BAP1‐competent cells (Figure 2A–D) and lipotoxicity is closely associated with lipid metabolism (Mathiowetz and Olzmann 2024), we hypothesized that inhibiting lipid metabolism may sensitize BAP1‐mutant UM. To test this, we treated BAP1‐competent (92.1 and Mel202) and BAP1‐mutant cells (MP65 and MP46) with increasing doses of atorvastatin (Ato, 0, 1, 10, and 25 μM), an inhibitor of HMG‐CoA reductase (Poli 2007), and measured cell proliferation using crystal violet staining. Atorvastatin treatment significantly reduced cell proliferation for BAP1‐mutant UM (Figures 3A, S6A–C), an effect that was associated with elevated lipid peroxidation (Figures 3B, S6D).

To further explore our in vitro studies in a more physiologically relevant model, we designed an ex vivo organotypic liver slice culture model to study liver metastatic UM. Here, 2.0 × 10^6^ GFP luciferase‐expressing MP46 cells (BAP1‐mutant) were injected into the spleens of NOD.Cg‐Prkdc^scid^ Il2rg^tm1Wjl^/SzJ (NSG) mice to facilitate hepatic UM seeding (similar to Sugase et al. 2020). Tumor burden was monitored by bioluminescent imaging for 3 weeks; livers were perfused and then harvested for organotypic liver slice cultures (Figure 3C). As expected, intrasplenic injection of UM cells resulted in colonization/“metastatic spread” to the liver parenchyma (Figure S7A). UM‐containing liver slices were then used to assess the therapeutic efficacy of Atorvastatin ex vivo (Figure 3C). Consistent with our in vitro studies, we found that Atorvastatin treatment significantly reduced UM tumor burden in a dose‐dependent manner (Figure 3D). Importantly, ATP measurements indicate that Atorvastatin treatment did not affect liver slice viability—highlighting a UM‐specific vulnerability (Figure S7B).

### 3.4 BAP1‐Mutant UM Cells Demonstrate Disruption of the BAP1/ASXL2 De‐Ubiquitination Complex

**FIGURE 4:**
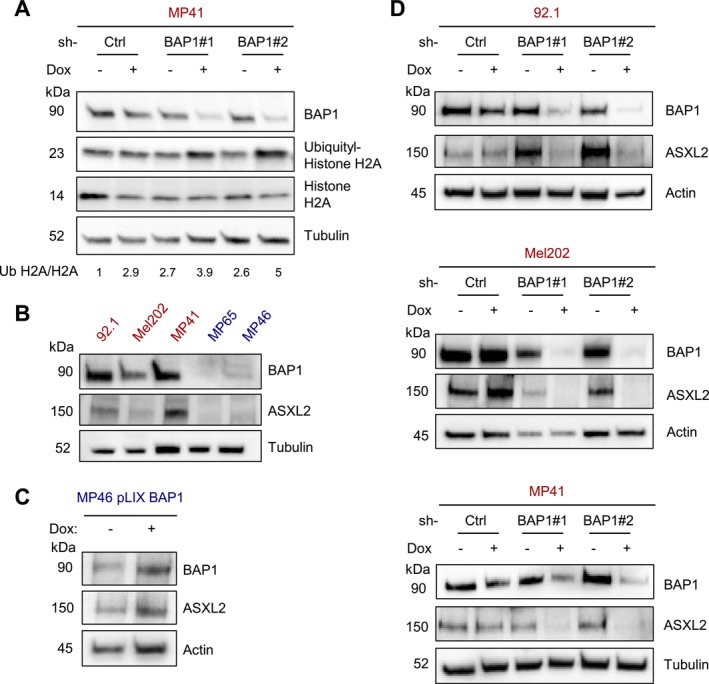
BAP1 loss is correlated with increased histone 2A ubiquitination and reduced ASXL2 expression. (A) Western blot of BAP1‐competent (MP41) cells after inducible shRNA‐mediated knockdown of BAP1 probing for BAP1, ubiquityl‐histone H2A, and Histone H2A expression with tubulin as a loading control. The ratio for relative intensity of ubiquityl‐histone H2A over Histone H2A levels is located below. (B) Western blot analysis of BAP1‐competent (92.1, Mel202, MP41) cells and mutant (MP65 and MP46) cells probing for BAP1 and ASXL2 expression at baseline with tubulin as a loading control. (C) Western blot analysis after inducible re‐expression of BAP1 in BAP1‐mutant (MP46) probing for ASXL2 and BAP1 with actin as a loading control. (D) Western blot analysis probing for ASXL2 expression upon inducible knockdown of BAP1 in BAP1‐competent (92.1, Mel202, and MP41) cells with actin and tubulin as loading controls. Western blots are representative of at least three independent repeated experiments.

To better understand how BAP1 loss may confer resistance to lipid‐induced stress, we explored links between BAP1 and lipid metabolism. BAP1 forms complexes with Additional Sex Combs Like‐2 (ASXL2) (Daou et al. 2015)—a polycomb group protein known to modulate lipid metabolism (Izawa et al. 2015; Park et al. 2014). The BAP1/ASXL2 complex regulates protein deubiquitination, particularly against histone H2A, thus affecting chromatin remodeling and gene expression (Daou et al. 2015; Peng et al. 2021; Sahtoe et al. 2016). However, the role of the BAP1/ASXL2 complex in UM is unknown. Using BAP1‐competent UM cell lines engineered to reduce BAP1 expression through doxycycline‐induced shRNA expression, we found that reduction of BAP1 correlated with increased histone H2A ubiquitination, suggesting that in UM, BAP1 contributes to epigenetic regulation (Figure 4A). Next, since previous studies demonstrate that BAP1 stabilizes ASXL2 (Daou et al. 2015, 2018), we conducted western blot analysis to compare baseline expression levels of ASXL2 and BAP1 in our human UM cell line panel. Notably, BAP1‐mutant UM have reduced ASXL2 expression (Figure 4B). To further explore the association between BAP1 and ASXL2 levels, we found that exogenous, doxycycline‐induced re‐expression of BAP1 in BAP1‐mutant UM cells (MP46) resulted in a concomitant increase in ASXL2 expression (Figure 4C). Conversely, inducible BAP1 depletion in BAP1‐competent cells (92.1, Mel202, and MP41) reduced ASXL2 (Figure 4D). Of note, in silico analysis of mutant BAP1 UM transcriptomic datasets (GSE78033 and TCGA) did not highlight any obvious correlation between BAP1 and ASXL2 expression—further supporting the role of BAP1 in stabilizing ASXL2 at the protein level (Daou et al. 2015). These findings suggest a direct relationship between BAP1 and ASXL2 expression in UM; and given ASXL2's role in lipid homeostasis, it may provide a rationale linking BAP1 status and lipid sensitivity.

### 3.5 ASXL2 Depletion Rescues Lipotoxicity Resistance

**FIGURE 5:**
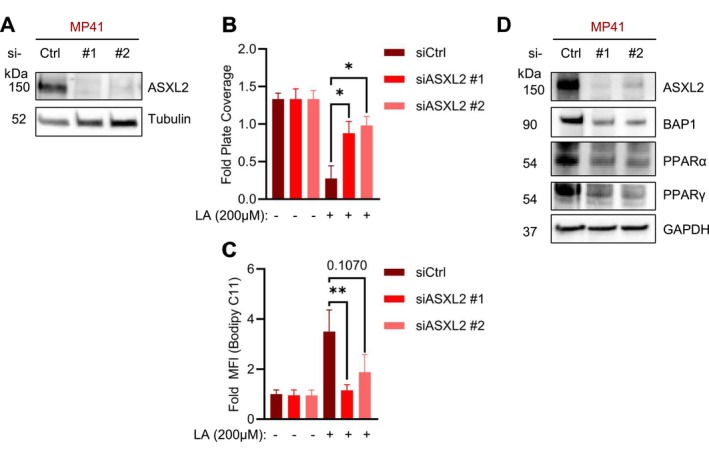
Altered lipid metabolism is due to the disruption of BAP1/ASXL2 in BAP1‐mutant UM. (A) Western blot of BAP1‐competent (MP41) cells after siRNA‐mediated knockdown of ASXL2 probing for ASXL2 with tubulin as a loading control. (B) Crystal violet quantification of BAP1‐competent (MP41) cells after siRNA knockdown of ASXL2 and incubation for 6 days treated with increasing levels of LA (0 and 200 μM). The quantification of crystal violet staining is represented by fold plate coverage compared to vehicle treatment. Representative crystal violet images can be found in Figure S8. (C) Lipid peroxidation levels indicated by Bodipy 493/593 C11 staining of MP41 cells treated following siRNA‐mediated knockdown of ASXL2 with and without treatment with 200 μM LA for 24 h. (D) Western blot of BAP1‐competent (MP41) cells after siRNA‐mediated knockdown of ASXL2 probing for ASXL2, BAP1, PPARα, PPARγ with GAPDH as a loading control. Western blots are representative images, and results are the averages from at least three independent repeated experiments. The * is indicative of *p* < 0.05, ** of *p* < 0.01, and # *p* < 0.0001 as determined by one‐way ANOVA analysis with multiple comparisons (B).

Given the positive correlation between BAP1 and ASXL2 and the role of ASXL2 in lipid homeostasis (Izawa et al. 2015; Park et al. 2014), we investigated how reduced levels of ASXL2 affect lipid tolerance in BAP1‐competent UM cells. First, we confirmed the efficacy of siRNA‐mediated ASXL2 knockdown in BAP1‐competent UM cells (MP41) (Figure 5A). Subsequently, we used cell proliferation assays to test if ASXL2 depletion alone could provide lipotoxicity resistance. Indeed, we found that reduced ASXL2 affords resistance to LA‐mediated toxicity (Figures 5B, S8), indicating that loss of ASXL2 can phenocopy lipid tolerance observed in BAP1‐mutant UM cells (Figure 2A,B).

Next, we investigated whether the enhanced proliferation observed under LA treatment following ASXL2 depletion correlates with changes in lipid peroxidation. Our analysis revealed that ASXL2‐depleted cells exhibited reduced lipid peroxide accumulation when exposed to LA compared to control cells, suggesting that ASXL2 plays a critical role in regulating cellular responses to lipid‐induced oxidative stress (Figure 5C).

Moreover, ASXL2 is known to interact with and activate PPARs, nuclear receptors involved in the regulation of triglyceride and lipid metabolism (Park et al. 2011; Gervois et al. 2000). Western blot analysis of BAP1‐competent cells (MP41) revealed reduced expression of PPARα and PPARγ when ASXL2 was reduced via siRNA (Figure 5D). These results suggest that BAP1‐mutant cells have increased lipid tolerance due to dysregulation of PPAR‐mediated lipid metabolism via reduced ASXL2 function.

## 4 Discussion

Our study reveals that BAP1 loss, present in 80% of liver metastatic UM lesions (Carbone et al. 2013), confers a selective advantage for survival in the lipid‐rich liver microenvironment through modulation of lipid tolerance and oxidative stress responses. This finding provides insight into why BAP1 mutations are strongly associated with hepatic metastasis in UM while also revealing a potential therapeutic vulnerability. Specifically, we highlight how BAP1‐mutant UM are largely resistant to lipotoxicity (Figure 2A,B)—an effect likely mediated through lipid peroxidation pathways (Figure 2C–F). Furthermore, we show that atorvastatin treatment reverses this phenotype (Figure 3), an observation that suggests clinical relevance, as statins are widely used and well‐characterized with established safety profiles (Thrift et al. 2019). Moreover, understanding how BAP1 status influences metabolic adaptation may inform the development of new therapeutic strategies for UM patients.

Furthermore, our work uncovers a role for BAP1's gene regulatory function in UM through its interaction with ASXL2, a key regulator of lipid homeostasis (Figure 4). The relationship between BAP1 and ASXL2 has been previously documented in other contexts, where they form a complex that regulates histone H2A deubiquitination and chromatin remodeling. In the present study, our findings implicate this complex contributes to lipid tolerance and builds upon emerging evidence suggesting that epigenetic regulators can influence lipid metabolism. Prior studies have shown that ASXL2 regulates lipid homeostasis; notably, ASXL2‐deficient mice develop lipodystrophy due to reduced PPARγ activity (Park et al. 2011; Izawa et al. 2015). Our current observation that ASXL2 depletion phenocopies the lipid tolerance of BAP1‐mutant cells (Figure 5) suggests conservation of this regulatory axis in UM and provides a mechanistic framework for understanding how BAP1 loss promotes disease progression through metabolic adaptation.

The BAP1/ASXL2 axis appears to influence PPAR expression, as ASXL2 depletion reduces PPAR levels in BAP1‐competent UM (Figure 5C). While we observe this association between BAP1/ASXL2 status and PPAR expression, the precise mechanisms linking this axis to lipid metabolism in UM warrant further investigation. To this end, PPAR signaling has been shown to regulate GPX4 expression and interface with chromatin modifiers like the NCOR/HDAC3 complex (Gervois et al. 2000; Xing et al. 2022; Kang and Fan 2020). Notably, BAP1 can regulate NCOR1 through deubiquitination, suggesting potential connections between epigenetic regulation and metabolic control (Yu et al. 2018).

Our current work likely has therapeutic implications. A current FDA‐approved therapy for metastatic UM includes Tebentafusp, a bispecific T‐cell engager that recruits CD3+ T cells to gp100‐expressing melanoma and has shown modest survival benefits (Hassel et al. 2023; Nathan et al. 2021). Previous studies have implicated immunogenic cell death in enhancing anti‐tumor T‐cell responses and pair well with immunotherapy approaches (Erkes et al. 2019; Rosenbaum et al. 2021; Niu et al. 2022; Wang et al. 2019). Eliciting lipid‐peroxide‐based cell death in mutant BAP1 UM is likely immunogenic as it parallels key aspects of ferroptosis. Therefore, it is possible that the efficacy of Tebentafusp could be augmented by combining it with agents that induce lipid‐peroxidation/ferroptotic cell death, such as atorvastatin.

While exploring therapeutic opportunities linked to BAP1/ASXL2‐mediated resistance to oxidative stress, our ex vivo liver slice model represents a platform to dissect complex mechanisms of metabolic adaptation and metastatic progression. Preservation of liver architecture and cellular diversity makes the system ideal for examining how lipid‐mediated metabolic rewiring shapes immune evasion mechanisms and treatment resistance. Furthermore, integrating spatial metabolomics and single‐cell profiling could reveal how metabolic heterogeneity within metastatic lesions influences therapeutic response. Understanding these complex interactions in a physiologically relevant context will be crucial for developing more effective treatments for metastatic uveal melanoma.

**FIGURE 6:**
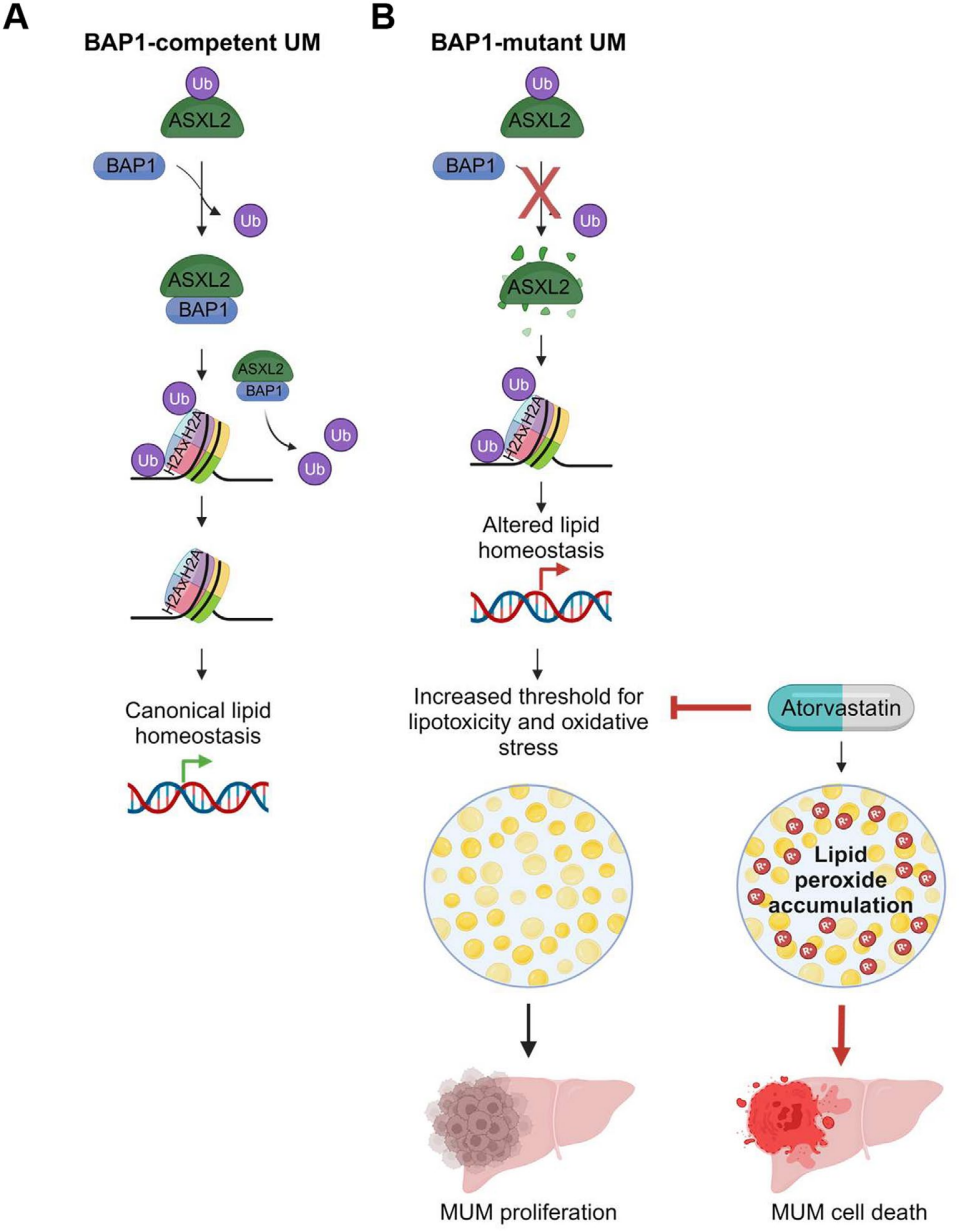
BAP1/ASXL2 complex loss promotes lipotoxicity resistance in BAP1‐mutant uveal melanoma. (A, B) Schematic representation of the BAP1/ASXL2 axis in UM. (A) In BAP1‐competent cells, BAP1 deubiquitinates and stabilizes ASXL2. The resulting BAP1/ASXL2 complex deubiquitinates Histone H2A, enabling canonical regulation of lipid homeostasis through proper gene expression. (B) In BAP1‐mutant cells, the absence of BAP1‐mediated stabilization leads to ASXL2 degradation. This disruption alters histone modification patterns and subsequent gene expression, resulting in dysregulated lipid homeostasis. The metabolic adaptation increases the threshold for lipotoxicity and oxidative stress, thereby facilitating proliferation in the lipid‐rich liver microenvironment. Importantly, atorvastatin treatment targets this metabolic vulnerability by inhibiting HMG‐CoA reductase, inducing lipid peroxide accumulation and selective MUM cell death, providing a potential therapeutic strategy for BAP1‐mutant liver metastases.

In conclusion, our study reveals a novel mechanism by which BAP1 loss in UM cells leads to adaptations that favor survival and proliferation in the lipid‐rich liver microenvironment. BAP1 loss in UM leads to ASXL2 reduction and lipid homeostasis dysregulation, conferring lipotoxicity resistance and an enhanced oxidative stress response—an adaptation that can be exploited with atorvastatin, which induces lipid peroxidation and cell death specifically in BAP1‐mutant liver metastases (Figure 6). Importantly, we demonstrate a selective vulnerability of BAP1‐mutant cells to agents that induce lipid peroxidation, suggesting a new therapeutic avenue for patients suffering from hepatic UM metastases.

## Author Contributions

**C. J. Cunanan:** conceptualization, investigation, writing – original draft, methodology, validation, visualization, writing – review and editing, formal analysis, data curation. **A. Amirfallah:** visualization, investigation, methodology, validation, formal analysis. **A. B. Sanders:** conceptualization, investigation, methodology, validation, visualization, formal analysis, data curation. **K. C. Gallant:** writing – original draft, writing – review and editing, visualization, validation, formal analysis, data curation. **M. R. Cavallo:** conceptualization, methodology, writing – review and editing. **E. A. Homer:** methodology, conceptualization. **O. S. El Naggar:** writing – original draft, writing – review and editing, methodology. **J. K. Farnan:** conceptualization, methodology. **G. Romano:** resources, conceptualization. **J. L. Hope:** conceptualization, resources, methodology. **J. G. Jackson:** conceptualization, methodology, resources. **E. J. Hartsough:** conceptualization, investigation, funding acquisition, writing – original draft, methodology, validation, visualization, writing – review and editing, supervision, data curation, formal analysis, resources, project administration.

## Disclosure

The authors have nothing to report.

## Supporting information


Table S1.

**Figure S1.** BAP1‐mutant UM patient samples are associated with elevated fatty acid metabolism. (A) Analysis of TCGA dataset looking at upregulated genes identified in the significantly upregulated fatty acid metabolism and phospholipase activity pathways in ClueGo analysis of GSE78033 (ASCL1, ACSS3, HMGCL, OXCT1, HACD1, HACD4, HSD17B12, ANXA1, ANXA2, ANXAP2, and ANXA4). Patient samples were stratified into BAP1‐competent versus BAP1‐mutant patient samples. The BAP1‐mutant group includes patient samples with either BAP1‐inactivating mutations or deletion. The * is indicative of *p* < 0.05, ** of *p* < 0.01, *** of *p* < 0.001, and # *p* < 0.0001 as determined using Welch’s *t*‐test.

**Figure S2.** BAP1‐mutant UM are more resistant to LA‐induced lipotoxicity. (A–C) Quantification (A), representative images (B) and summary table of statistically significant differences between BAP1 competent versus mutant cell lines (C) of crystal violet staining of 92.1, Mel202, MP41, MP65, and MP46 cells treated with increasing levels of LA (0, 50, 100, 200, and 300 μM) for 6 days as indicated by crystal violet staining. The quantification of crystal violet staining is represented by fold plate coverage compared to vehicle treatment. Results are the averages from at least three independent repeated experiments. The ns is indicative of *p* > 0.05, * of *p* < 0.05, ** of *p* < 0.01, *** of *p* < 0.001, and # *p* < 0.0001 as determined by two‐way ANOVA analysis with multiple comparisons (A, C).

**Figure S3.** BAP1‐mutant UM are more resistant to DHA‐induced lipotoxicity. (A, B) Quantification (A), representative images (B) and summary table of statistically significant differences between BAP1 competent versus mutant cell lines (C) of crystal violet staining of 92.1, Mel202, MP41, MP65, and MP46 cells treated with increasing levels of DHA (0, 25, 50, 100, 150 μM) for 6 days. The quantification of crystal violet staining is represented by fold plate coverage compared to vehicle treatment. Results are the averages from at least three independent repeated experiments. The ns indicative of *p* > 0.05, * of *p* < 0.05, ** of *p* < 0.01, *** of *p* < 0.001, and # *p* < 0.0001 as determined by two‐way ANOVA analysis with multiple comparisons (A, C).

**Figure S4.** BAP1‐mutant UM are more resistant to LA and DHA‐induced lipid peroxidation. (A, B) Table representing statistically significant differences between cell lines in Bodipy 493/593 C11 staining treated with 0, 200, or 300 μM LA for 24 h (A) or 150 μM DHA for 96 h (B). Bodipy 493/593 C11 staining is represented by fold MFI compared to vehicle treatment. (C, D) Cell proliferation indicated by crystal violet staining (D) and lipid peroxidation levels indicated by Bodipy 493/593 C11 (E) of 92.1 cells treated with 2 μM Lip1, 300 μM LA, or both. Results are the averages from at least three independent repeated experiments. The ns indicative of *p* > 0.05, * of *p* < 0.05, ** of *p* < 0.01, *** of *p* < 0.001, and # *p* < 0.0001 as determined by two‐way ANOVA analysis with multiple comparisons (A, B).

**Figure S5.** LA‐induced lipotoxicity is not rescued by pan‐caspase inhibition. (A, B) Representative images (A) and quantification (B) of crystal violet staining of MP41 cells treated with 10 μM ZVADFMK, 300 μM LA, or both for 96 h. Quantification of crystal violet staining is represented by fold plate coverage compared to vehicle treatment. Results are the averages from at least three independent repeated experiments. The ns indicative of *p* > 0.05, * of *p* < 0.05, ** of *p* < 0.01, *** of *p* < 0.001, and # *p* < 0.0001 as determined by two‐way ANOVA analysis with multiple comparisons.

**Figure S6.** BAP1‐mutant UM are sensitive to lipid metabolism inhibition. (A, B) Quantification (A), representative images (B), and summary table of statistically significant differences between BAP1 competent versus mutant cell lines (C) of crystal violet staining of 92.1, Mel202, MP65, and MP46 cells treated with increasing levels of Ato (0, 1, 5, 10, 25 μM) for 6 days. Quantification of crystal violet staining is represented by fold plate coverage compared to vehicle treatment. (D) Table representing statistically significant differences between BAP1 competent versus mutant cell lines in Bodipy 493/593 C11 staining treated with increasing levels of Ato (0, 25, and 50 μM) for 24 h. Bodipy 493/593 C11 staining is represented by fold MFI compared to vehicle treatment. Results are the averages from at least three independent repeated experiments. The ns indicative of *p* > 0.05, * of *p* < 0.05, ** of *p* < 0.01, *** of *p* < 0.001, and # *p* < 0.0001 as determined by two‐way ANOVA analysis with multiple comparisons.

**Figure S7.** Ex vivo model of liver UM metastases remains viable under Ato treatment. (A) Representative confocal images of ex vivo model of BAP1‐deficient (MP46‐GFP) liver metastatic UM stained with anti‐CD31 (Red) under 10× and 30× objective. (B) Viability of organotypic liver slice culture indicated by ATP levels treated with Ato (0 and 80 μM) for 72 h. Results are the averages from at least three independent repeated experiments with 2–6 slices per group with *n* = 3 mice. The ns indicative of *p* > 0.05, * of *p* < 0.05, ** of *p* < 0.01, *** of *p* < 0.001, and # *p* < 0.0001 as determined by two‐way ANOVA analysis with multiple comparisons.

**Figure S8.** ASXL2 siRNA knockdown rescues LA‐induced lipotoxicity. A. Representative images of crystal violet staining of BAP1‐competent (MP41) after siRNA knockdown of ASXL2 followed by incubation for 6 days with increasing levels of LA (0 and 200 μM).

## Data Availability

The publicly available data analyzed in this study are from GSE78033 and UVM‐TCGA datasets. The analyzed data for these datasets are available in Table S1.
