# Supplementary figures and images for: BAP1 Loss Affords Lipotoxicity Resistance in Uveal Melanoma
Source: Pigment Cell Melanoma Res. 2025 Apr 29;38(3):e70021. doi: 10.1111/pcmr.70021 (PMC12040534; doi:10.1111/pcmr.70021)

**A**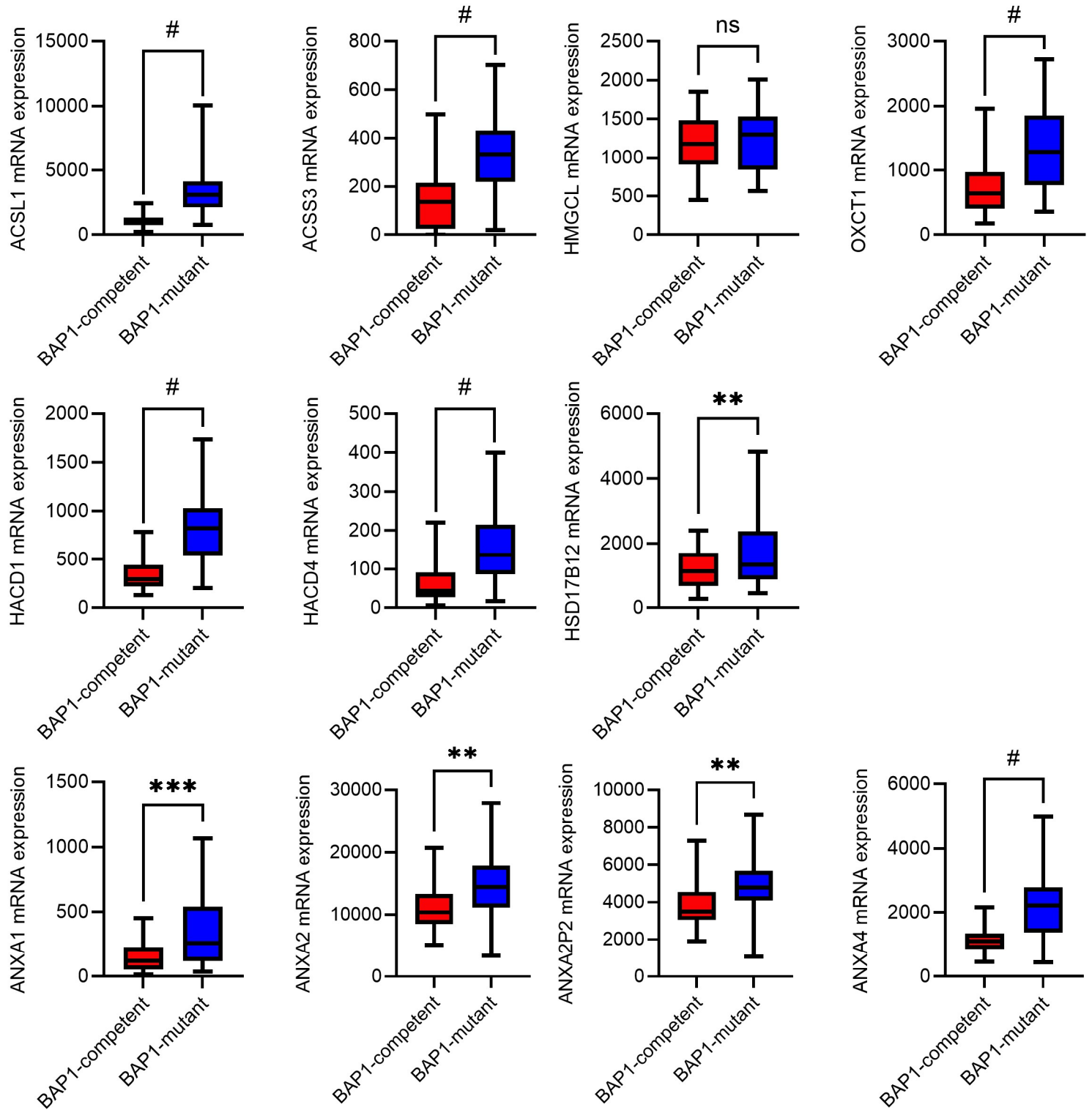

Supplement: Supplementary file 2 — Figure S1. BAP1‐mutant UM patient samples are associated with elevated fatty acid metabolism. (A) Analysis of TCGA dataset looking at upregulated genes identified in the significantly upregulated fatty acid metabolism and phospholipase activity pathways in ClueGo analysis of GSE78033 (ASCL1, ACSS3, HMGCL, OXCT1, HACD1, HACD4, HSD17B12, ANXA1, ANXA2, ANXAP2, and ANXA4). Patient samples were stratified into BAP1‐competent versus BAP1‐mutant patient samples. The BAP1‐mutant group includes patient samples with either BAP1‐inactivating mutations or deletion. The * is indicative of p < 0.05, ** of p < 0.01, *** of p < 0.001, and # p < 0.0001 as determined using Welch’s t‐test. [file PCMR-38-0-s005.pdf]

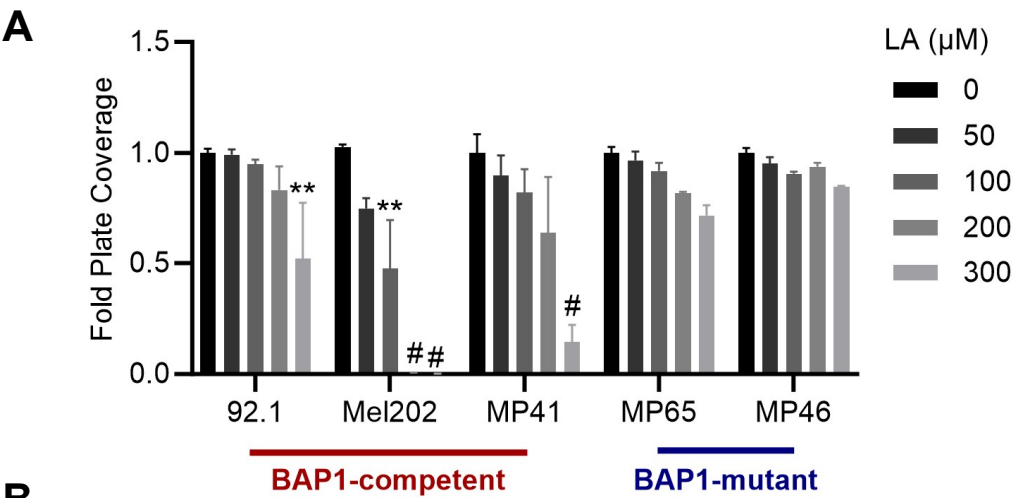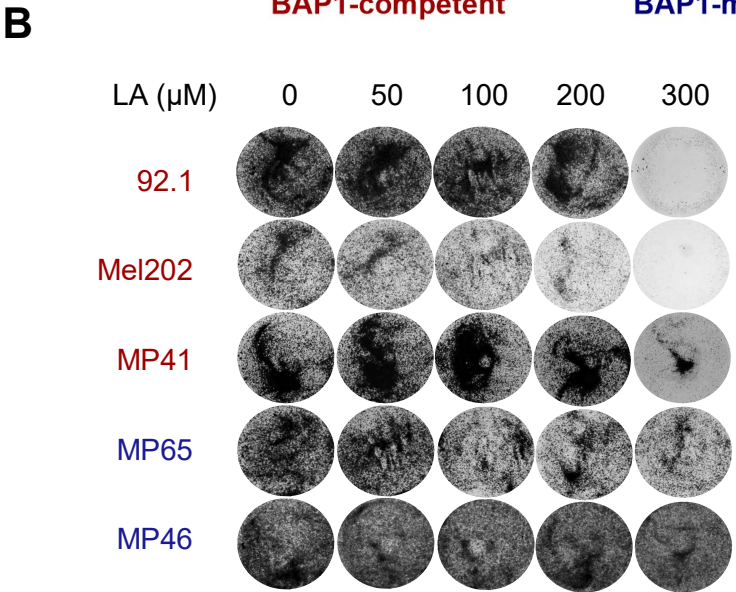

**C**

|                      | MP65 |    |     |     |     | MP46 |    |     |     |     |
|----------------------|------|----|-----|-----|-----|------|----|-----|-----|-----|
| LA ( $\mu\text{M}$ ) | 0    | 50 | 100 | 200 | 300 | 0    | 50 | 100 | 200 | 300 |
| 92.1                 | ns   | ns | ns  | ns  | ns  | ns   | ns | ns  | ns  | ns  |
| Mel202               | ns   | ns | *   | #   | #   | ns   | ns | *   | #   | #   |
| MP41                 | ns   | ns | ns  | ns  | **  | ns   | ns | ns  | ns  | #   |

Supplement: Supplementary file 3 — Figure S2. BAP1‐mutant UM are more resistant to LA‐induced lipotoxicity. (A–C) Quantification (A), representative images (B) and summary table of statistically significant differences between BAP1 competent versus mutant cell lines (C) of crystal violet staining of 92.1, Mel202, MP41, MP65, and MP46 cells treated with increasing levels of LA (0, 50, 100, 200, and 300 μM) for 6 days as indicated by crystal violet staining. The quantification of crystal violet staining is represented by fold plate coverage compared to vehicle treatment. Results are the averages from at least three independent repeated experiments. The ns is indicative of p > 0.05, * of p < 0.05, ** of p < 0.01, *** of p < 0.001, and # p < 0.0001 as determined by two‐way ANOVA analysis with multiple comparisons (A, C). [file PCMR-38-0-s002.pdf]

**A**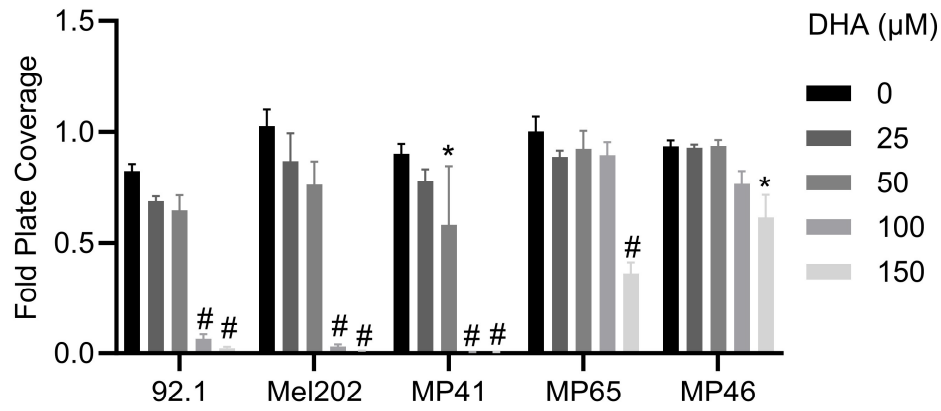**B**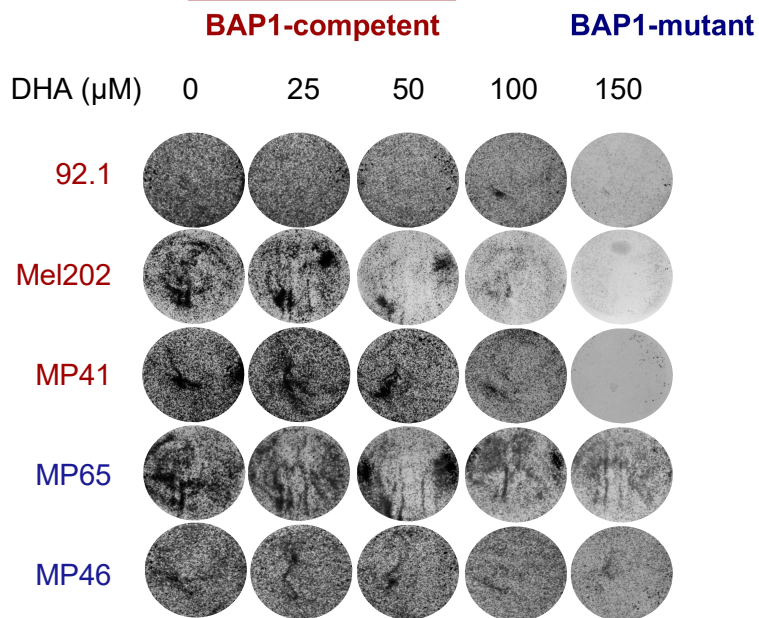**C**

|          | MP65 |    |    |     |     | MP46 |    |    |     |     |
|----------|------|----|----|-----|-----|------|----|----|-----|-----|
| DHA (μM) | 0    | 25 | 50 | 100 | 150 | 0    | 25 | 50 | 100 | 150 |
| 92.1     | ns   | ns | ns | #   | *   | ns   | ns | ns | #   | #   |
| Mel202   | ns   | ns | ns | #   | *   | ns   | ns | ns | #   | #   |
| MP41     | ns   | ns | *  | #   | *   | ns   | ns | *  | #   | #   |

Supplement: Supplementary file 4 — Figure S3. BAP1‐mutant UM are more resistant to DHA‐induced lipotoxicity. (A, B) Quantification (A), representative images (B) and summary table of statistically significant differences between BAP1 competent versus mutant cell lines (C) of crystal violet staining of 92.1, Mel202, MP41, MP65, and MP46 cells treated with increasing levels of DHA (0, 25, 50, 100, 150 μM) for 6 days. The quantification of crystal violet staining is represented by fold plate coverage compared to vehicle treatment. Results are the averages from at least three independent repeated experiments. The ns indicative of p > 0.05, * of p < 0.05, ** of p < 0.01, *** of p < 0.001, and # p < 0.0001 as determined by two‐way ANOVA analysis with multiple comparisons (A, C). [file PCMR-38-0-s008.pdf]

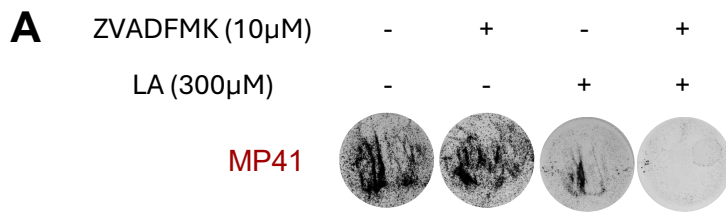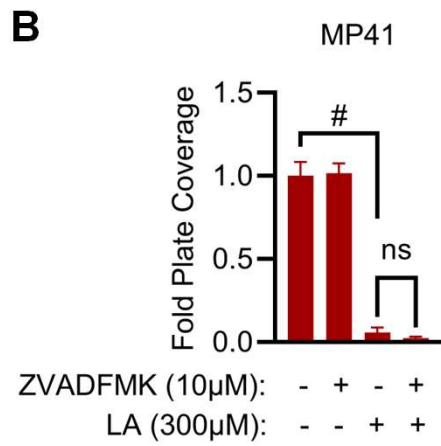

Supplement: Supplementary file 6 — Figure S5. LA‐induced lipotoxicity is not rescued by pan‐caspase inhibition. (A, B) Representative images (A) and quantification (B) of crystal violet staining of MP41 cells treated with 10 μM ZVADFMK, 300 μM LA, or both for 96 h. Quantification of crystal violet staining is represented by fold plate coverage compared to vehicle treatment. Results are the averages from at least three independent repeated experiments. The ns indicative of p > 0.05, * of p < 0.05, ** of p < 0.01, *** of p < 0.001, and # p < 0.0001 as determined by two‐way ANOVA analysis with multiple comparisons. [file PCMR-38-0-s001.pdf]

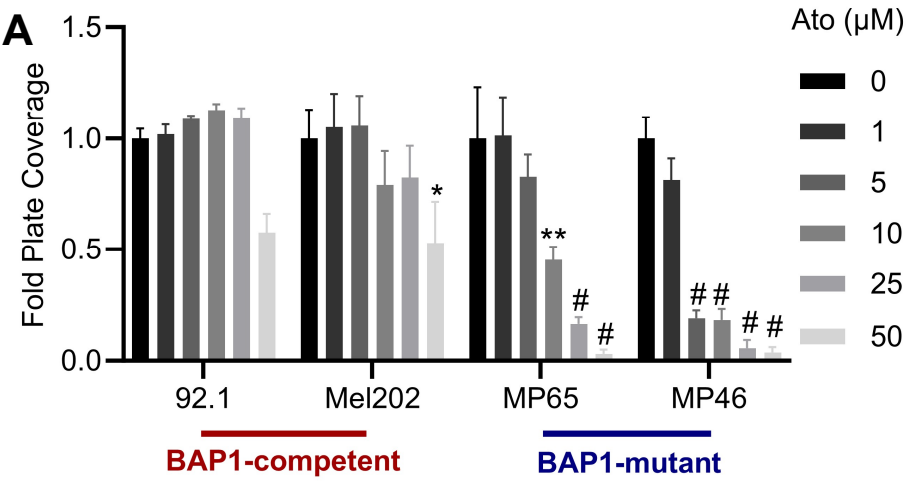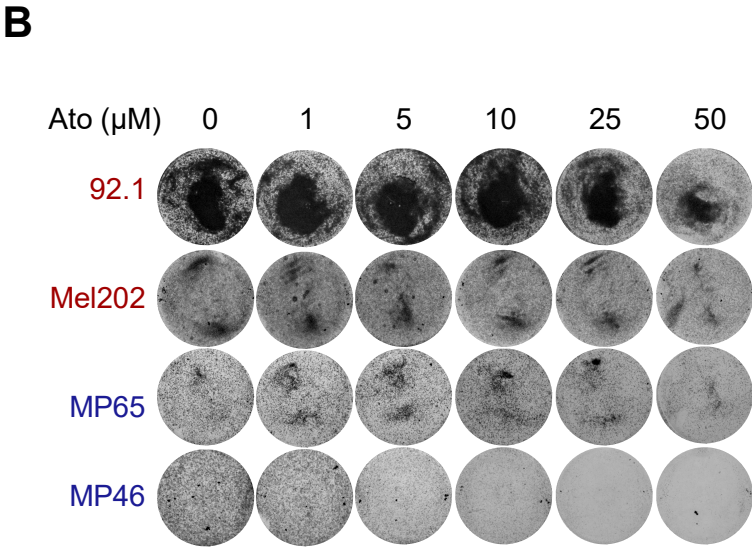

**C**

|                       | MP65 |    |    |     |     |    | MP46 |    |   |    |    |    |
|-----------------------|------|----|----|-----|-----|----|------|----|---|----|----|----|
| Ato ( $\mu\text{M}$ ) | 0    | 1  | 5  | 10  | 25  | 50 | 0    | 1  | 5 | 10 | 25 | 50 |
| 92.1                  | ns   | ns | ns | *** | #   | ** | ns   | ns | # | #  | #  | ** |
| Mel202                | ns   | ns | ns | ns  | *** | ** | ns   | ns | # | ** | #  | *  |

**D**

|                       | MP65 |    |    | MP46 |    |    |
|-----------------------|------|----|----|------|----|----|
| Ato ( $\mu\text{M}$ ) | 0    | 25 | 50 | 0    | 25 | 50 |
| 92.1                  | ns   | ns | ** | ns   | ns | ns |
| Mel202                | ns   | ns | #  | ns   | ns | *  |

Supplement: Supplementary file 7 — Figure S6. BAP1‐mutant UM are sensitive to lipid metabolism inhibition. (A, B) Quantification (A), representative images (B), and summary table of statistically significant differences between BAP1 competent versus mutant cell lines (C) of crystal violet staining of 92.1, Mel202, MP65, and MP46 cells treated with increasing levels of Ato (0, 1, 5, 10, 25 μM) for 6 days. Quantification of crystal violet staining is represented by fold plate coverage compared to vehicle treatment. (D) Table representing statistically significant differences between BAP1 competent versus mutant cell lines in Bodipy 493/593 C11 staining treated with increasing levels of Ato (0, 25, and 50 μM) for 24 h. Bodipy 493/593 C11 staining is represented by fold MFI compared to vehicle treatment. Results are the averages from at least three independent repeated experiments. The ns indicative of p > 0.05, * of p < 0.05, ** of p < 0.01, *** of p < 0.001, and # p < 0.0001 as determined by two‐way ANOVA analysis with multiple comparisons. [file PCMR-38-0-s009.pdf]

**A**

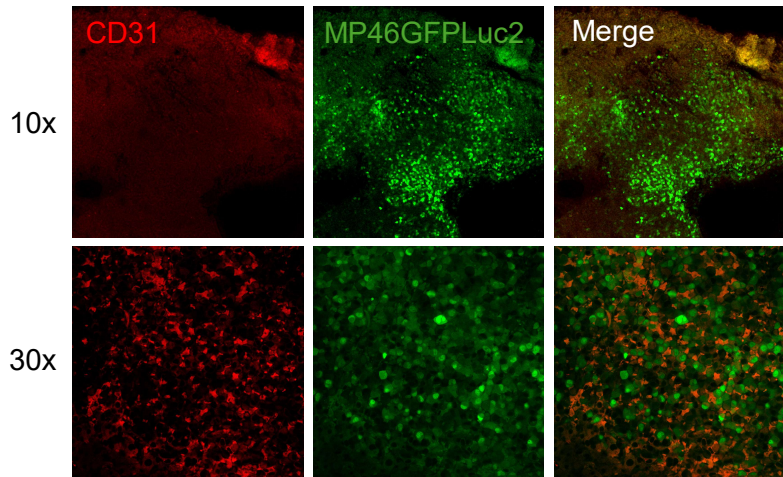

# B

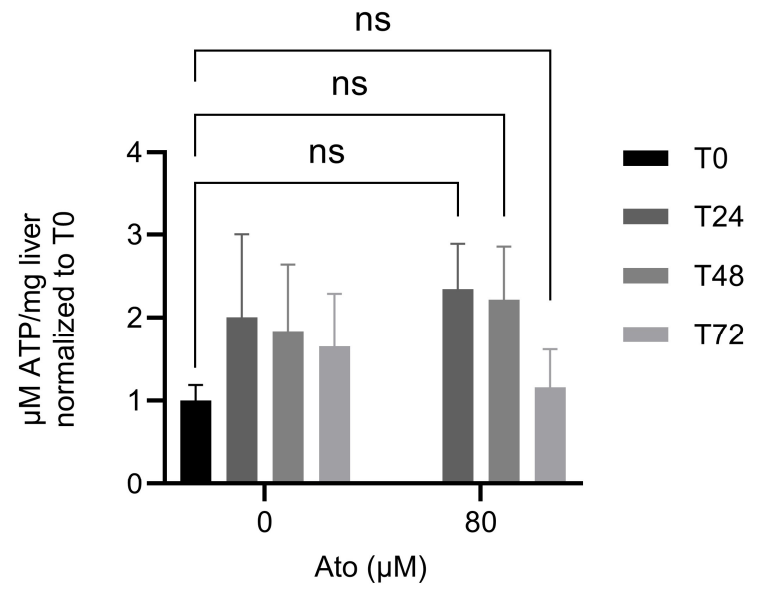

Supplement: Supplementary file 8 — Figure S7. Ex vivo model of liver UM metastases remains viable under Ato treatment. (A) Representative confocal images of ex vivo model of BAP1‐deficient (MP46‐GFP) liver metastatic UM stained with anti‐CD31 (Red) under 10× and 30× objective. (B) Viability of organotypic liver slice culture indicated by ATP levels treated with Ato (0 and 80 μM) for 72 h. Results are the averages from at least three independent repeated experiments with 2–6 slices per group with n = 3 mice. The ns indicative of p > 0.05, * of p < 0.05, ** of p < 0.01, *** of p < 0.001, and # p < 0.0001 as determined by two‐way ANOVA analysis with multiple comparisons. [file PCMR-38-0-s006.pdf]

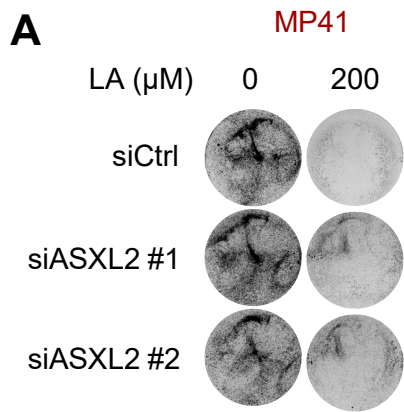

Supplement: Supplementary file 9 — Figure S8. ASXL2 siRNA knockdown rescues LA‐induced lipotoxicity. A. Representative images of crystal violet staining of BAP1‐competent (MP41) after siRNA knockdown of ASXL2 followed by incubation for 6 days with increasing levels of LA (0 and 200 μM). [file PCMR-38-0-s007.pdf]
