# Supplementary material for: BAP1 Loss Affords Lipotoxicity Resistance in Uveal Melanoma
Source: Pigment Cell Melanoma Res. 2025 Apr 29;38(3):e70021. doi: 10.1111/pcmr.70021 (PMC12040534; doi:10.1111/pcmr.70021)

A

	MP65			MP46		
LA (µM)	0	200	300	0	200	300
92.1	ns	ns	ns	ns	ns	ns
Mel202	ns	ns	*	ns	ns	***
MP41	ns	ns	ns	ns	ns	ns

B

	MP65		MP46	
DHA (µM)	0	150	0	150
92.1	ns	ns	ns	ns
Mel202	ns	ns	ns	ns
MP41	ns	#	ns	#

C

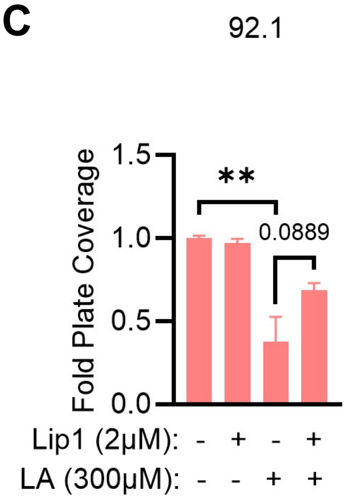

D

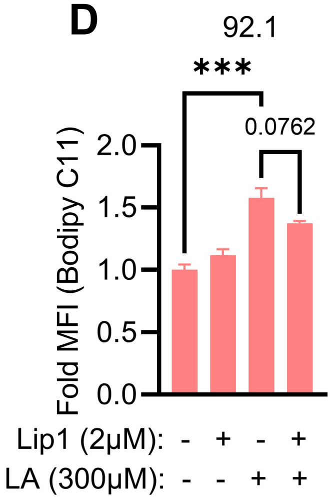

Supplement: Supplementary file 5 — Figure S4. BAP1‐mutant UM are more resistant to LA and DHA‐induced lipid peroxidation. (A, B) Table representing statistically significant differences between cell lines in Bodipy 493/593 C11 staining treated with 0, 200, or 300 μM LA for 24 h (A) or 150 μM DHA for 96 h (B). Bodipy 493/593 C11 staining is represented by fold MFI compared to vehicle treatment. (C, D) Cell proliferation indicated by crystal violet staining (D) and lipid peroxidation levels indicated by Bodipy 493/593 C11 (E) of 92.1 cells treated with 2 μM Lip1, 300 μM LA, or both. Results are the averages from at least three independent repeated experiments. The ns indicative of p > 0.05, * of p < 0.05, ** of p < 0.01, *** of p < 0.001, and # p < 0.0001 as determined by two‐way ANOVA analysis with multiple comparisons (A, B). [file PCMR-38-0-s004.pdf]
